# Assessment of pulmonary antibodies with induced sputum and bronchoalveolar lavage induced by nasal vaccination against *Pseudomonas aeruginosa: *a clinical phase I/II study

**DOI:** 10.1186/1465-9921-8-57

**Published:** 2007-08-05

**Authors:** Ulrich Baumann, Kerstin Göcke, Britta Gewecke, Joachim Freihorst, Bernd Ulrich von Specht

**Affiliations:** 1 Department of Paediatric Pulmonology and Neonatalogy, Hanover Medical School, Carl-Neuberg Str. 1, 30625 Hannover, Germany; 2 Pediatric Hospital, Ostalb-Klinikum, 73430 Aalen, Germany; 3 Centre for Clinical Research, Freiburg University, Breisacherstr.66, 79106 Freiburg, Germany

## Abstract

**Background:**

Vaccination against *Pseudomonas aeruginosa *is a desirable albeit challenging strategy for prevention of airway infection in patients with cystic fibrosis. We assessed the immunogenicity of a nasal vaccine based on the outer membrane proteins F and I from *Pseudomonas aeruginosa *in the lower airways in a phase I/II clinical trial.

**Methods:**

N = 12 healthy volunteers received 2 nasal vaccinations with an OprF-OprI gel as a primary and a systemic (n = 6) or a nasal booster vaccination (n = 6). Antibodies were assessed in induced sputum (IS), bronchoalveolar lavage (BAL), and in serum.

**Results:**

OprF-OprI-specific IgG and IgA antibodies were found in both BAL and IS at comparable rates, but differed in the predominant isotype. IgA antibodies in IS did not correlate to the respective serum levels. Pulmonary antibodies were detectable in all vaccinees even 1 year after the vaccination. The systemic booster group had higher IgG levels in serum. However, the nasal booster group had the better long-term response with bronchial antibodies of both isotypes.

**Conclusion:**

The nasal OprF-OprI-vaccine induces a lasting antibody response at both, systemic and airway mucosal site. IS is a feasible method to non-invasively assess bronchial antibodies. A further optimization of the vaccination schedule is warranted.

## Background

Prevention of chronic airway infection with *Pseudomonas aeruginosa *is a major goal in therapy of cystic fibrosis (CF) patients. We and others developed vaccines for use in CF based on various pseudomonal antigens, including lipopolysaccharides, toxin A, flagella, alginate, and outer membrane proteins [1-4]. Our vaccine antigen is a recombinant fusion protein of the highly conserved outer membrane proteins OprF and OprI from *P. aeruginosa*. The OprF-OprI vaccine was shown to afford protection in various animal models and to be safe and immunogenic in several clinical trials [5-7]. In an attempt to enhance the formation of antibodies at the airway surface, the site of the *P. aeruginosa *infection in CF, we pursued a nasal vaccination strategy. Nasal vaccination is known to specifically induce an antibody response of the bronchus-associated lymphoid tissue (BALT) resulting in an enhanced at the upper and lower airways [8,9]. The nasal OprF-OprI gel vaccine was well tolerated and elicited a reliable systemic immune response in experimental and clinical studies [4,10,11].

The present study continues the work on the nasal OprF-OprI gel vaccine. Our aims were to assess the antibody formation at the pulmonary airway surface, to assess the persistence of antibody levels after one year, and to compare two vaccination schedules.

Assessment of antibodies in the human lower airways raises the question of the appropriate method. Vaccine induced pulmonary antibodies have been obtained by bronchoalveolar lavage (BAL) [9,12] However, BAL is a relatively invasive measure preventing its use in larger clinical trials. Moreover, BAL fluid (BALF) has a predominantly alveolar site of origin and may not adequately represent the antibody composition at the bronchial surface. This prompted us to investigate whether the well-established technique of induced sputum (IS) is a way to reliably assess antibodies from the bronchial airways. IS is used for diagnostic procedures in a number of airway diseases, including CF and chronic obstructive pulmonary disease (COPD), in both children and adults [13,14]. We evaluated the feasibility of the IS technique for assessment of bronchial antibodies in comparison to BAL.

The second aim was to assess the antibody systemic and mucosal antibody response not only immediately following immunization, but also after one year. The kinetics of mucosal antibody formation may not necessarily have similar kinetics as the systemic antibody response due to their differential induction and regulation mechanisms [8].

Finally, we compared two variants of nasal vaccination schedules. We investigated whether the immunogenicity of the nasal OprF-OprI vaccine can be enhanced by a systemic booster vaccination. A systemic booster vaccination was effective in augmenting the mucosal antibody response to the oral polio live vaccine [15].

The present study establishes IS as a valuable method to obtain antibodies from the bronchial surface not represented by BAL. The nasal OprF-OprI engendered a lasting systemic and mucosal immune response irrespective of the booster schedule.

## Methods

### Production of the Vaccines

The nasal and systemic OprF-OprI vaccines were produced as described [6,10]. Briefly, the hybrid protein (Met-Ala-[His]_6 _OprF190-342-OprI21-83) consisting of the mature outer membrane protein I (OprI) and amino acids 190–342 of OprF of *P. aeruginosa*, was expressed in *E. coli *and purified by Ni2^+ ^chelate-affinity chromatography [5]. For the nasal vaccine, an aqueous solution of the OprF-OprI protein was emulsified into a gel containing 1% OprF-OprI, 45% sodium dodecyl sulfate (Merck, Darmstadt, Germany), and 5% aerosil (Caesar and Lorenz, Hilden, Germany). The final concentration was 10 mg OprF-OprI/ml. For the systemic vaccine, OprF-OprI protein was adsorbed to aluminum hydroxide (Superfos, Vedbaeck, Denmark) and diluted into normal saline, together with thimerosal (Caesar and Lorenz, Hilden, Germany) as a preservative. The final concentrations were 0.1 mg OprF-OprI/ml, 0.3 mg Al(OH)_3_/ml and 0.05 mg thimerosal/ml.

### Subjects

12 male, healthy subjects (mean age 24.3, range 21 to 26 years) participated in the study. Exclusion criteria were any chronic condition, such as diseases of the nose, immune deficiencies, a previous *P. aeruginosa *infection, or regular use of drugs. The volunteers gave a detailed medical history and underwent a physical examination prior to the study, including examination of the nasal cavity by an ENT-specialist. Total IgG and IgA levels in serum and IgA levels in saliva were obtained in order to exclude an as yet unknown humoral immune defect prior to inclusion in the study.

All subjects gave written informed consent. The study was in accordance to the Helsinki declaration, approved by the ethics committee of Hanover Medical School, and registered with the German authority for vaccines and sera (Paul-Ehrlich Institute, Langen, Germany).

### Study Design

All subjects received a nasal primary followed by a nasal booster vaccination (day 0 and 21, respectively). At day 42, the second booster was given either nasally (n = 6, "nasal booster group"), or systemically (n = 6, "systemic booster group"). The participants were assigned to the schedule by drawing lots to ensure equal numbers for both groups. For nasal vaccination, 100 μl of the emulgel, containing 1 mg OprF-OprI, were placed on the cranial side of the middle concha nasalis by a 24 gauge Teflon cannula. Systemic vaccination was performed by injection of 1 ml, containing 100 μg OprF-I, into the deltoid muscle.

Serum and induced sputum (IS) were obtained 1 day prior to the primary vaccination (day -1), at day 70, i.e. 4 weeks after the second booster vaccination, and after 1 year. Bronchoalveolar lavage (BAL) was performed prior to the first vaccination and 4 weeks after the second booster vaccination. BAL was performed 24 hrs after the IS sampling procedure.

Data on tolerability and the mean values of OprF-OprI specific antibody levels in serum at weeks 0 and 10 were published previously [11].

### Surveillance

The vaccinees were observed clinically for 4 h after each vaccination and after the BAL procedures. The body temperature was obtained prior to and 1, 4, 12, 24, 48, and 72 hrs after each vaccination. The local and systemic responses were graded on a scale from 0 to 3, with the respective scores representing absent, mild, moderate, and severe reactions. Reactions to the vaccine were assessed for 3 consecutive days and documented by the volunteers. In addition, each volunteer underwent a physical examination 2 days after each vaccination. Blood samples were drawn prior to the vaccination, as well as 2 and 14 days after each vaccination. The samples were assessed for full blood count, evaluation of liver enzymes, creatinine, and urea.

BAL was performed in accordance to the ERS taskforce recommendations [16]. Briefly, a flexible bronchoscope (BF P40, Olympus, Hamburg, Germany) was used for instillation of 5 × 20 ml of normal saline with the bronchoscope placed in the middle lobe bronchus in wedge position. BAL samples were immediately placed on ice and subsequently centrifuged at 60 × g and 4 for 10 min. For analysis of the cellular content, cytospots were performed with 200 μl of native BAL at 55 × g for 10 minutes and stained with May Grünwald/Giemsa

Enumeration of the cytospots revealed normal cellular proportions in all samples, with mean proportions as follow: macrophages, 90.1 ± 4.7 (SD)%; lymphocytes, 8.0 ± 4.8%; and neutrophils, 1.3 ± 0.7%). The recovery rates were 36 ± 5.2% and 43 ± 3.8% in the first and second BAL procedures, respectively. Analysis of BAL fluid from one subject in the nasal booster group was excluded due to a haemorrhagic sample.

### Induced Sputum

Sputum induction was performed in accordance to the ERS guidelines with the exception of use of 5.85% saline solution (Braun, Melsungen, Germany) [17]. Briefly, inhalation time was 2 min, followed by oral cleansing with water, drying of the mouth, and subsequent sputum expectoration over a period of 3 min. The procedure was repeated five times. Sputum samples were collected separately, immediately placed on ice, and stored at -80°C. The recovered volumes of IS were 6.0 ± 0.8 ml and 13.2 ± 1.3 ml in the first and second sampling, respectively.

### Development of the Method for Antibody Detection in Sputum

The use of IS for antibody detection had to be evaluated prior to the study, as the procedure was not described in the literature. We used sputum expectorated by CF patients with chronic *P. aeruginosa *infection. As IS is commonly mixed with dithiothretiol (DTT) to separate its cellular and mucous contents, we first tested whether antibody detection in sputum was either improved or impaired by this additive. Sputum specimens from 3 CF patients who gave written informed consent for use of the specimen were gently mixed 1:1 with a working solution of 0.1% DTT in normal saline (Calbiochem, Bad Soden, Germany) according to the manufacturer's instructions, or with normal saline. The specimens were subsequently centrifuged at 4 and 17,000 × g for 30 min. The supernatants showed similar levels of OprF-OprI specific antibody levels in ELISA irrespective of the use of either DTT or NaCl 0.9%. DTT was therefore not used further for sputum antibody analysis.

Next, we analyzed whether high-speed centrifugation would improve the rate of antibody extraction. Sputum was centrifuged at 4 either for 4 h at 120,000 × g, or for 30 min at 17,000 × g. The antibody levels in supernatants were comparable irrespective of the centrifugation technique. We therefore used conventional centrifugation.

Finally, we investigated whether anti proteases would improve the extraction rate of antibodies from the sputum of CF patients. We added 2 μl 0.5 M ethylene diamine tetraacetic acid (EDTA) and 1 μl of a 100 mM phenyl methyl sulfonyl fluoride (PMSF) solution in isopropanol to 100 μl of unprocessed CF – sputum, in order to inhibit the activity of metalloproteases and serine proteases, respectively. Supernatants of sputa treated with protease inhibitors showed OprF-OprI-specific IgG and IgA antibody levels that were two- to threefold higher than those of untreated specimens. Protease inhibitors were therefore used for the study. It remains, however, unclear whether the addition of protease inhibitors was necessary, since the IS of the healthy volunteers is likely to have minimal protease content compared to sputum from chronically infected CF patients.

### Blood Samples

Blood samples were stored overnight at 4 without anticoagulants and centrifuged at 7,800 × g for 10 min. The supernatant was frozen at -80 until analysis.

### ELISA assays

96-well microtiter plates were coated with OprF-OprI at 1 μg/ml, blocked by a 0.2% bovine serum albumin (BSA, Sigma, Munich, Germany) solution, and incubated with 100 μl/well of serum diluted at 1:2,000, with unconcentrated BAL, or with supernatant of sputum diluted at 1:2 for 2 h at 37. Binding was visualized with peroxidase-conjugated IgG- or IgA-specific binding rabbit antihuman secondary antibodies (Dako, Hamburg, Germany, the Binding Site, Birmingham, UK), diluted 1:10,000, with tetra methyl benzidine (TMB, Sigma-Aldrich, Munich, Germany) as the chromogen. After 30 min of incubation at room temperature, the reaction was stopped with 1 M sulfuric acid. The extinction was read at λ = 450 nm. All assays were performed in duplicate. Antibody levels are given as arbitrary ELISA units (EU) based on the optical density (OD) multiplied by the dilution factor and normalized against an internal standard serum. An immune response to the vaccination was considered positive only, if the EU was more 50% above the individual pre-vaccination value.

### Statistics

Mean values are given together with the standard error of the mean (SEM). Mean values of independent groups were compared by two-sided Student's *t*-test following the estimation of equality of the variance by Levene's test or by paired-samples *t*-tests, as appropriate. Correlations were calculated with the product moment correlation coefficient (Pearson's coefficient). Linear regression analysis was used to calculate a regression line, with ELISA units in serum as independent variables, and in BAL or IS as dependent variables.

A p value of less than 0.05 was considered significant. Calculations were performed with the SPSS program V.14 (SPSS Inc., Chicago, IL, USA).

## Results

### Systemic antibody response

**Figure 1:**
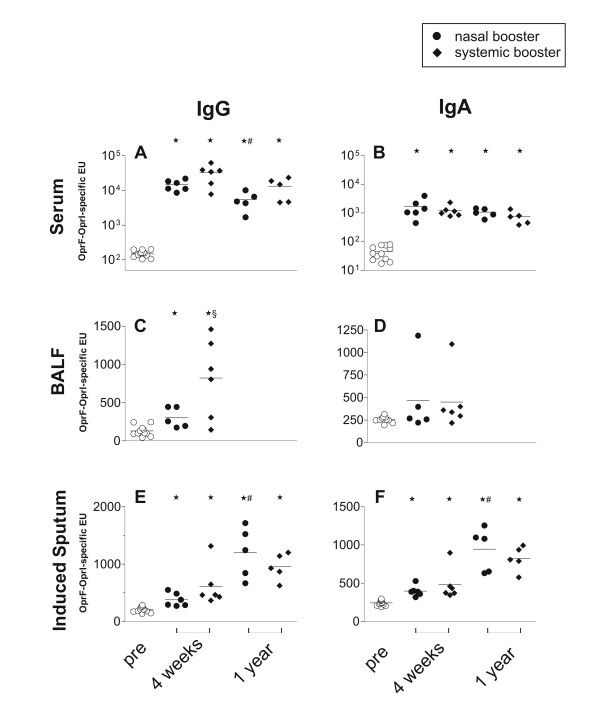
Individual OprF-OprI-specific IgG (left column) and IgA (right columns) antibody levels in ELISA units in serum (upper panel), pooled bronchoalveolar lavage fluid (BALF, middle panel, and supernatant of induced sputum (IS, lower panel) prior to the vaccination (open circles), 4 weeks and 1 year after the booster vaccination. N = 12 volunteers received 2 applications with 1 mg of a nasal OprF-OprI gel vaccine, followed by either a third nasal vaccination (nasal booster group, closed circles, n = 6), or by a systemic vaccination with 100 μg OprF-OprI protein (systemic booster group, closed diamonds, n = 6). Mean values are indicated by the line. Mean values of the groups were compared with the paired, two-sided t-test and a p value of less than 0.05 considered significant. * assign significant differences of mean values to the pre-vaccination values, # between mean values at 4 weeks and 1 year, and §between the vaccination groups. For clarity of the figure, pre vaccination levels of both vaccination groups are shown together.

All vaccinees had a pronounced and lasting immune response with formation of IgG and IgA antibodies in serum irrespective of the the vaccination protocol (Figure 1A, and 1B, Table 1). After 1 year, antibody levels appeared to drop moderately. However, this reached statistical significance only in for IgG antibodies in the nasal booster group. The nasal booster group showed also a trend (p = 0.05) towards lower levels at 4 weeks compared to the systemic booster group (Figure 1A).

**Table 1:** Responder rates

**IgG**	**4 weeks**		**1 year**	
	**nasal booster**	**systemic booster**	**nasal booster**	**systemic booster**
**Serum**	6/6	6/6	6/6	6/6
**BALF**	4/5	6/6	n.a.	n.a.
**Induced Sputum**	3/6	5/6	5/5	5/5
				
**IgA**	**4 weeks**		**1 year**	
	**nasal booster**	**systemic booster**	**nasal booster**	**systemic booster**

Serum	6/6	6/6	6/6	6/6
**BALF**	1/5	3/6	n.a.	n.a.
**Induced Sputum**	4/6	3/6	5/5	5/5

Responder rates for OprF-OprI-specific IgG (left part of the table) and IgA (right part) antibody formation in serum, pooled bronchoalveolar lavage fluid (BALF), and supernatant of induced sputum (IS) 4 weeks and 1 year after the vaccination. For vaccination schedule refer to Figure legend 1. An antibody response was considered positive, if the post-vaccination ELISA unit was 50% or more above the individual pre-vaccination level. Data are given numbers of individuals with positive response vs. the total number of vaccinees of this group. n.a. = not applicable, since BAL was performed at 4 weeks only.

### Mucosal antibody response

At 4 weeks, specific antibodies were detectable in the majority of subjects in both, BALF and IS (Figure 1C–F, Table 1). In BALF, however, antibodies were predominantly of IgG isotype. The systemic booster group had significantly higher levels of IgG antibodies than the nasal booster group. In contrast to the serum antibody response, antibodies in IS were higher at 1 year than at 4 weeks, reaching statistical significance in the nasal booster group for both isotypes (Figure 1E, and 1F).

### Sequential analysis of BALF and IS

**Figure 2:**
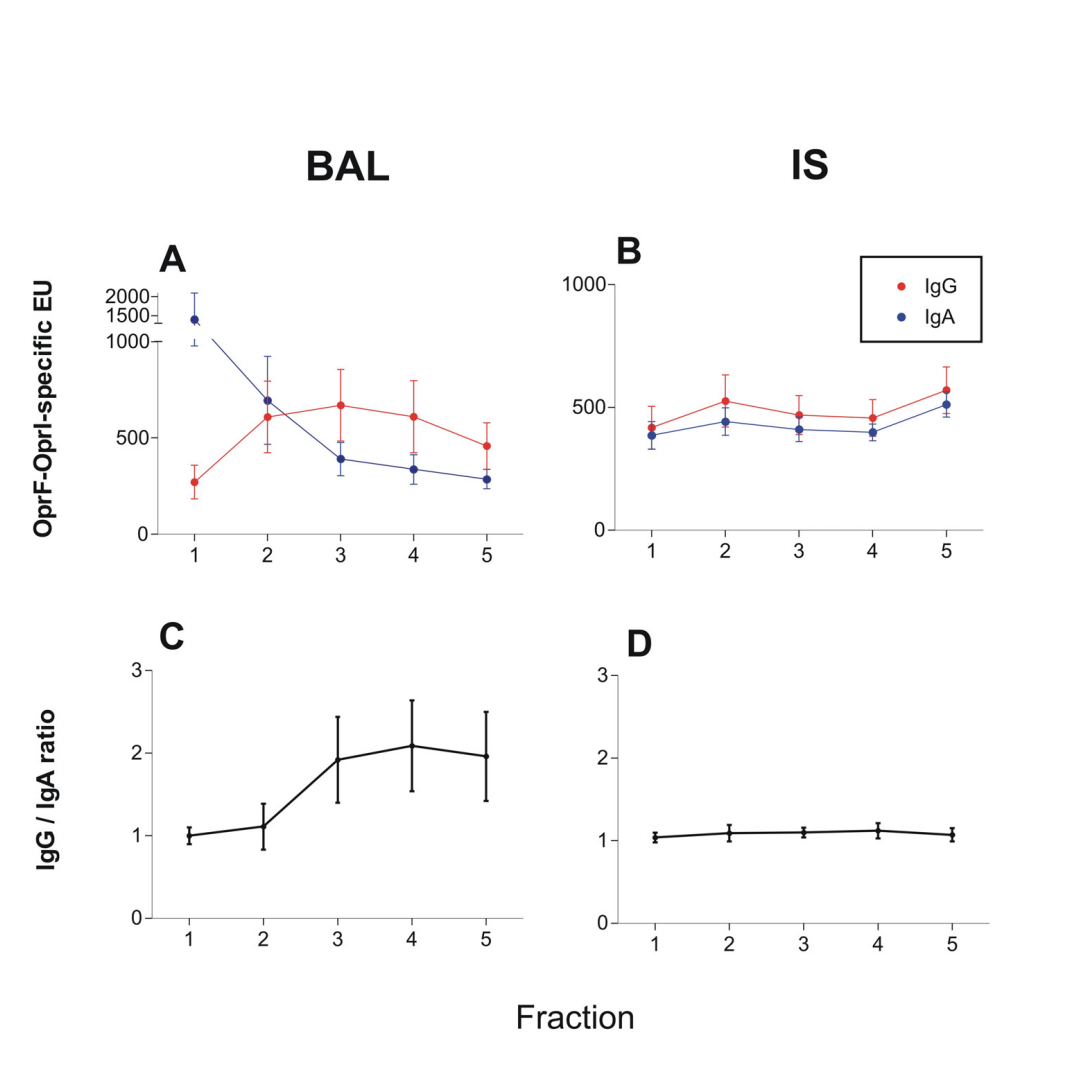
Sequential analysis of OprF-OprI-specific IgG and IgA antibodies in single fractions of bronchoalveolar lavage fluid (BALF, A and C) and induced sputum (IS, B and D) of n = 12 healthy volunteers vaccinated with nasal primary and a nasal or systemic booster vaccination. Specimen were obtained 4 weeks after the booster vaccination. The upper panel shows the antibody levels in ELISA units, the lower panel the ratio of the IgG to the IgA levels. Data are given as values (circles) and standard error of the mean (SEM, lines). Antibody composition changed significantly between the first 2 and the consecutive samples in BAL, while it remained stable in all fractions in IS. Mean values of ratios were compared by two-sided paired t-test. A p-value of less than 0.05 was considered significant. * indicate significant differences to the first 2 fractions.

Analysis of the single fractions of BALF showed an increase of IgG levels and concomitantly a decrease of IgA levels, resulting in an increase of the IgG to IgA ratio (figure 2A, and 1C). In IS, however, IgG to IgA antibody levels and their ratio remained largely unchanged in all fractions (Figure 2B, and 2D). The first two fractions of BALF were indifferent in their IgG to IgA ratios to the ratios of IS.

### Correlation of systemic and mucosal antibody levels

**Figure 3:**
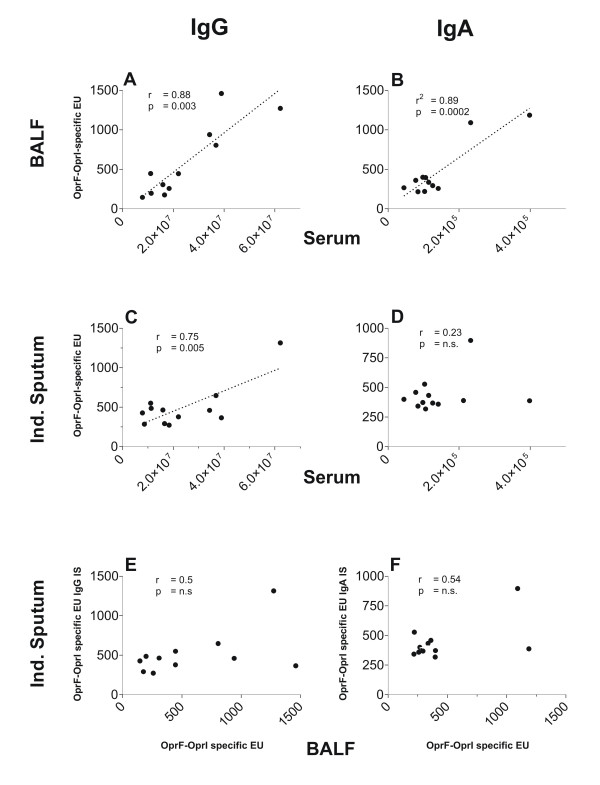
Individual levels of OprF-OprI-specific IgG (left colms) and IgA values (right column) given as correlation between bronchoalveolar lavage fluid (BALF) and serum (upper panel), between induced sputum and serum (middle panel) and between induced sputum and BALF (lower panel) obtained 4 weeks after completion of the vaccination schedule in n = 12 volunteers. Lines represent linear regression in cases of where correlation is significant. Pearson's product moment correlation cofficients of correlation (r) and levels of significance (p) are given as numerical data in each section.

Antibody levels in BALF correlated strongly to the related serum levels with both isotypes (Figure 3A, and 3B). There was also a correlation between antibody levels of IS and serum, albeit more weakly and restricted to the IgG isotype. IgA antibodies in IS were not correlated to IgA levels in serum. Antibodies in IS and BALF showed no significant correlation (Figure 3E, and 3F).

## Discussion

This study shows that the nasal OprF-OprI vaccine induces a immune response not only in serum, but also in the lower airways. The observation that antibodies were detectable in both compartments after 1 year at high levels suggest a lasting immunogenicity and a high responder rate of this anti pseudomonal vaccination strategy. Assessment of pulmonary antibodies showed a comparable sensitivity of BAL and IS. However, the different proportions of the isotypes suggest that the two methods represent at least in part different areas of the lower airways. The origin of fractional BAL samples is known to advance from the bronchial to the alveolar compartment of the lower airways [18]. Accordingly, we found high concentrations of IgG antibodies, the predominant isotype in the alveolar space, in the 3^rd ^to the 5^th ^BAL fraction, and higher IgA concentrations, the predominant isotype on the bronchial mucosa, in the1st and 2^nd ^BAL fraction [19]. The IgG to IgA ratio in IS was comparable to the composition of first 2 BAL fractions. Studies comparing cellular components obtained with BAL and IS support a differential origin of the samples with IS deriving from the central airways [14,20]. This suggests that the antibody composition on the bronchial mucosa is relatively stable over larger parts of the bronchial tree.

IgG antibody levels in serum correlated well to the levels in BALF and, albeit more weakly, in IS. IgG is thought to reach the alveoli by passive diffusion from the systemic circulation, and subsequently move by mucociliary transport towards the central airways [19]. IgG in the bronchial airways may then be complemented by antibodies produced locally as suggested by the presence of IgG positive antibody secreting cells in the lamina propria [21]. The weak correlation of IgG antibodies in IS to serum IgG is consistent with this dual origin of bronchial IgG antibodies.

IgA antibody levels in IS showed no correlation to systemic and BALF antibody levels of this isotype. These findings are consistent with a bronchial origin of the antibodies regulated differently than the systemic immunity [8]. Bronchial IgA predominantly derives from antibody secreting cells located in the lamina propria of the bronchial mucosa and not from the systemic circulation [21].

The present study is, to our knowledge, the first to use IS for assessment of vaccine induced antibodies. The relatively homogeneous results suggest that the method is technically feasible and applicable in individuals who do not expectorate spontaneous sputum. But is this method clinically meaningful? The answer obviously depends on the condition addressed by a vaccine. In CF, *P. aeruginosa *infection affects predominantly the bronchial mucosa [22]. A strong immunogenicity at the bronchial mucosa, therefore, may be particularly desirable for anti pseudomonal vaccines for patients with CF. With IS representing the bronchial antibody composition better than pooled BALF or serum, this technique may be usefully applied in the process of optimization of the vaccination strategy.

The use of IS for detection of vaccine induced antibodies has limitations. A major drawback of the IS technique is its age restriction [23]. Assessment of mucosal antibody response in infants and pre-school age children, the primary target group for CF, has to resort to other strategies, such as the detection of ASC with mucosal homing receptors. It has also to be noted that the presence of bronchial antibodies does not necessarily translate into protection.

In the present study we assessed the efficacy of a combined nasal primary systemic booster vaccination compared to a solely nasal vaccination schedule. The systemic booster vaccination induced a stronger and more lasting systemic IgG antibody response. A more frequent nasal vaccination as performed in the nasal booster group, however, was more efficient in the induction of a long term immune response at the bronchial mucosa with both IgG and IgA isotypes. Both schedules, therefore, do not appear ideal. Further research on mucosal and/or vaccination schedules is warranted.

A surprising finding was that antibody levels in IS were higher after 1 year than the post vaccination levels obtained at 4 weeks. A technical error such as a an inappropriate ELISA procedure seems unlikely as all assays used the same positive and negative control sera, and the concomitant assessment of serum antibodies did show the expected decrease in the 1 year specimen. However, mucosal vaccination and antibody formation may not necessarily follow the same kinetics. While is appears unlikely that mucosal antibody formation rises after 1 year, the first assessment at 4 weeks may have missed the peak of mucosal antibody formation.

The findings of the present study facilitate the further development of the OprF-OprI vaccine. Current studies compare antibody levels at the bronchial surface engendered by the systemic, nasal and a newly developed oral vaccine all based on the same antigen in healthy volunteers and in patients with chronic lung disease. It also remains to be shown whether the OprF-OprI vaccine can be effective in patients with CF and other conditions. Recent data of a phase III clinical trial with CF patients evaluating a bivalent flagella vaccine of *P. aeruginosa *suggest that a protein based vaccine can be protective in CF patients. However, protection was shown only against those *P. aeruginosa *strains that expressed a vaccine type of the flagella protein [24]. Our OprF-OprI based vaccine is based on an antigen which is cross-reactive against all serotypes of *P. aeruginosa *appears to be a promising candidate for further vaccine development aimed to protect patients with CF from chronic *P. aeruginosa *airway infection.

## Conclusion

We conclude that the nasal OprF-OprI vaccine induces a lasting antibody response in serum and the lower airways. Antibodies at the bronchial surface can be non-invasively assessed by IS. A systemic booster is not effective to further enhance the airway immunogenicity of the nasal OprF-OprI vaccine. Recent data of a phase III clinical trial with a flagella vaccine support both the promise of protein based vaccines and the need for further optimization of the vaccine strategy. Our present study which employs a vaccine with a broad cross-reactivity may facilitate further vaccine development by the use of the IS technique.

## Competing interests

UB and BuvS receive funding under a grant from the centre for innvation and technology (ZIT), Vienna, Austria, for a joint Pseudomonas vaccine project conducted together with an affiliate of Intercell AG, Vienna, Austria.

## Authors' contributions

UB lead and evaluated the study and wrote the manuscript; KG recruited the volunteers, assisted in the sampling procedures, and performed the ELISA measurements as part of her medical thesis; BG assisted at the ELISA procedures; JF performed the bronchoalveolar lavage; BUvS developed and produced the OprF-OprI vaccine and wrote the grant application; All authors read and approved the final manuscript.
